# Relational care and epistemic injustice

**DOI:** 10.1017/S1463423623000555

**Published:** 2023-10-23

**Authors:** Rupal Shah, Sanjiv Ahluwalia, John Spicer

**Affiliations:** 1 Health Education England, London, UK; 2 Anglia Ruskin University, Cambridge, UK; 3 Institute of Medical and Biomedical Education, St George’s University of London, London, UK

**Keywords:** autonomy, epistemic injustice, primary care

## Abstract

The philosophical underpinnings of primary care have been examined from several perspectives in recent years. In two previous articles, we have argued that a relational view of autonomy is better matched to the primary care setting than others, and that view is mainly formed from the descriptors of its practice. Here we develop that analysis further, linking it to other relevant theory: the experience of human suffering and epistemic injustice. We argue that relational care is fundamental to ameliorating epistemic injustice and that relationships are integral to ethical practice, rather than being distinct. We propose that personalised care as described in the NHS Long Term Plan is not possible without addressing epistemic injustice and therefore without reconsidering our existing normative ethical frameworks.

## Introduction

In this article, we apply a relational view of ethics specifically to people who are at high risk of epistemic injustice, building on two previous articles in which we explored challenges to respect for autonomy and the influence of complexity on moral choice (Spicer *et al.*, 2021; Spicer *et al.*, 2021). Miranda Fricker first described epistemic injustice as being injustice inflicted on someone in their capacity as a knower (Fricker, 2007). She described two components to this – hermeneutic injustice and testimonial injustice. Havi Carel and Ian Kidd went on to apply Fricker’s conceptualisation of epistemic justice to the experience of patients (Carel and Kidd, 2014). Testimonial injustice occurs when a patient’s account of how they experience illness is dismissed or under-played by the clinician, who selects those parts of the story which he or she considers to be ‘useful’ in allowing categorisation in the form of a diagnosis or in deciding a treatment route. Hermeneutic injustice occurs when a patient is unable to make sense of her symptoms and experiences because she is not able to articulate her story in a way that validates it – her experience is not represented within the medical lexicon. In such situations, patients are often viewed as unreliable narrators of their own stories and their experience is interpreted through a distorting medical lens. This in turn impacts the capacity for real decision-making that might emerge from such a clinical encounter. This is especially pertinent when the life experience of the patient is very far removed from that of the clinician.

Justice, in its wider principlist meaning, is described as a notion of equity, fairness, or even distributive justice. As such it depends on equity between persons in, for example, access to the advantages of health care. We draw a distinction between these versions of justice and those of Carel and Kidd *supra.*

The NHS Long Term Plan mandates that personalised care should become ‘business as usual’ across the health and care system by 2023/4 (NHS Long Term Plan, 2019). Key stated components of personalised care are shared decision-making and personalised care planning. We argue that neither of these are possible without redressing epistemic injustice; and further that personalised care is contingent on a relational view of ethics. This contrasts with the prevailing mindset within primary healthcare, which privileges population health by incentivising guideline-based management of disease, without considering the phenomenology of illness, that is, how it is experienced by patients.

## Illness and the creative self – the relevance of EI to personalised care

Illness can change the way in which people experience life, influencing identity and leading to a change in values and priorities (Carel, 2021). According to Carel, the act of processing personal trauma affords an opportunity for individuals to come to terms with the disruption that has occurred to their sense of reality and to consider what brings value to life in their new, altered existence. Carel has proposed that people have creative capacity to adapt in the face of illness and to re-imagine life in a new context (Carel, 2007) and Reeve further developed this concept by putting forward the idea of the ‘creative self’ who can adapt in response to internal and external stimuli (Reeve, 2017). Other authors have similarly proposed that patients have intrinsic capabilities or assets, which should be utilised by practitioners in co-creating personalised treatment plans (Sen, 1990; Ekman *et al.*, 2011).

Healthcare professionals, particularly those in primary care, have the potential to support the creative capacity and agency of their patients, mediated through the relationships they build up over time. However, as those of us working in primary care know, this does not always happen. When considering why not, the concept of epistemic injustice is relevant. If the meaning of the illness for the individual is missed, the creative capacity that Carel and others describe will not be harnessed and the individual’s decision-making is diminished. An example from everyday practice is obesity in the context of trauma and poverty. The standard approach of providing information and support around healthy choices is unlikely to be effective if the powerful underlying behavioural drivers are ignored, including amongst many other factors, the patient’s relationship to food. As Darren McGarvey states in his book ‘Poverty Safari’, ‘the thought of those first few bites, the emotional relief, and instant fulfilment they induce possess such an allure that resistance is futile’ (McGarvey, 2018: 115). Change is nevertheless possible but is more likely to be achieved by acknowledging and exploring the intense emotional response to food. A relational perspective of human-to-human interactions with a focus on understanding and being sensitive to the context of the patient is fundamental; as is trying to comprehend the meaning of the illness as it is experienced by the patient. Doing so increases agency and empowerment by supporting creative capacity; and is thus an antidote to epistemic injustice. Such relational and context-specific perspectives require healthcare professionals to exercise moral choice and to use their personal knowledge of their patients, whilst also acknowledging the non-universal nature of human experience.

## A relational ethical framework

A clarification of what we mean by relational care is apposite here. A rather functional view of primary care would subsume a patient interacting with a clinician to seek, and get, a ‘solution’ to the immediate problem: a course of treatment, advice about lifestyle or other. It is possible that recent developments in technology have accelerated such a transactional version of care – for example, triage systems that require people to input their presenting complaint and allow the clinician to respond without directly interacting with the patient, sometimes by text message.

However, interactions in primary care are often repeated over time, sometimes many years, and inevitably relationships develop between individual clinicians and their patients. Such relationships can be manifest in accessibility, trust and as is increasingly demonstrated, better health outcomes (Brown *et al.*, 2020). There is a wealth of descriptive and analytical scholarship on the nature and purpose of relationships in primary care, which we do not seek to challenge. Our purpose here is to consider the moral status of such relationships, and whether they can be used to address the issue of epistemic justice as we have described it.

Relational ethics perhaps suffers from being less clearly defined than other normative ethics. However, within a relational ethics framework, ethical decision-making is enmeshed within and dependent upon the practitioner-patient relationship. Its central tenets have been stated to be mutual respect, engagement, embodied knowledge, environment, and uncertainty (Pollard, 2015). Pollard argues that interactions between people generate a feeling of responsibility for the other and that it is this which determines the morality of the subsequent action. We agree with her view that the nature of the relationship itself has bearing on the morality or otherwise of clinical decisions – a relationship based on mutual respect and engagement enables human factors as well as biomedical aspects of illness to be factored into such decisions.

We apply this relational view of ethical decision-making to three hypothetical patients whose social context makes them particularly vulnerable to epistemic injustice. These cases are derived from our own professional experience as being useful illustrative examples and are composites.

## Clinical case 1

*A 48-year-old woman with type 2 diabetes, Ms Z, is offered the choice by her GP of being referred to a dietitian or of starting insulin to help with her sugar control, which is poor, despite the three oral hypoglycaemic medications she already takes. She lives alone and takes medication for depression. She works as a cashier and is on a zero-hours contract, which means it is hard to predict when she can make time for appointments; in any case, she doesn’t think a dietitian would help her. She would rather not take another medicine but chooses this option after the consultation with her GP.*

There is increasing evidence that diabetes and depression are syndemic. That is, they are co-occurring epidemics, which are both linked to underlying social factors such as trauma and poverty (Singer *et al.*, 2017; McGarvey, 2018). It is likely that treating these ‘upstream’ driving forces could significantly improve both the diabetes and the depression from which Mrs Z suffers – however, Mrs Z is unlikely to have been given this explanation during a standard consultation or options to mitigate against the social factors driving her illnesses. Because the underlying drivers of Mrs Z’s illnesses have not been addressed, she could be considered to be a victim of epistemic injustice within this consultation, even though the GP attempted to act ethically by following guidelines, weighing up the risks of benefit versus harm and by sensitively communicating the medical choices available to her and allowing her to make the final decision.

The problem arises because the choices offered to Mrs Z by her GP are generic and applicable to any patient with the same disease markers. Mrs Z’s individual circumstances have not been considered, including the effect of the burden of treatment (Sav *et al.*, 2015). It could be argued that Mrs Z’s health would be improved by action to tackle the underlying social difficulties, which exacerbate her physical and mental ill health. The impact of these difficulties could be exposed and addressed through the relationship between Mrs Z and her GP.

The notion of individual autonomy and therefore of empowerment to participate in shared decision-making is challengeable in any context (Ives *et al.*, 2018; Spicer *et al.*, 2021) but is particularly difficult in cases such as that of Mrs Z; and assuming that autonomy is independent of context and of the clinician-patient relationship may deepen epistemic injustice.

## Clinical case 2

*A 55-year-old woman, Mrs M, who is a refugee from Afghanistan is seen with an interpreter to discuss pain management. She complains of widespread body pain. She is offered the choice of a codeine/paracetamol mix or physiotherapy and is signed off work. Her relationship with her husband is very strained and her grown-up children have moved away from the family home. She has no extended family in the UK.*

‘Pain’ has a particular meaning when seen through a clinical lens. For Mrs M, there may be a large overlap with suffering, which has been missed. It is an example of a patient’s experience of illness not fitting easily into medical categories that are created by doctors. Without understanding Mrs M’s experience of pain, the options offered may not help her. If we accept that Mrs M has the potential to adapt in the face of illness using her creative capacity, it becomes the moral duty of a treating clinician to help her make sense of her new life circumstances. In fact, there is a clear link between relational ethics and person-centred care, which has already been established (Tomaselli, 2020) – harnessing Mrs M’s own creative capacity can only happen through relational care, probably consisting of multiple encounters over a period, with the focus being to try to understand her past experiences and to help her make the link between what has happened to her and her physical symptoms. In this case, although she is offered the choice between different biomedical interventions, she is nevertheless the victim of epistemic injustice. Any decision she makes cannot be fully informed or autonomous because she is not being offered options that are likely to relieve her suffering.

## Clinical case 3

*A 25-year-old woman, Ms T, has a diagnosis of emotionally unstable personality disorder. There is an alert on her notes warning about aggression and impulsive behaviour. There is also a code on her medical records of childhood sexual abuse. Quetiapine is prescribed when pregabalin alone fails to stabilise her mood.*

This is another example in which the creative capacity of the patient, Ms T, has not been supported. The presence of the alert may inhibit the formation of an engaged, authentic relationship founded on mutual respect between practitioner and patient, which allows Ms T to understand the link between her childhood trauma and her current problems. The prescription of medications with the potential for significant adverse effects is potentially unjust, even though they may help to manage some of the manifestations of Ms T’s distress. The prescribing clinician will have assessed her mental state, but in the absence of an on-going relationship will have been unable to understand her lived experience and the drivers for her behaviour. In other words, she is a victim of hermeneutic injustice.

Each of the protagonists in the case studies above is at risk of epistemic injustice and application of a normative ethical framework is unlikely to protect them. We argue that personalised care described in the NHS Long Term Plan as a statutory duty of healthcare providers is not possible without addressing epistemic injustice. Surfacing and articulating the moral ambiguity inherent in clinical decisions is not well captured by existing ethical frameworks. For this, an explicitly relational view is required, which incorporates influences on the decision-making of both clinician and patient and the nature of the relationship between clinician and patient.

Although primary care clinicians do not have the ability to solve the structural reasons for poverty and trauma, applying a relational model of ethics has significance in terms of addressing epistemic injustice. The nature of the relationship between practitioner and patient will determine the extent to which the patient’s experience of illness is understood and considered when making decisions; and thus, the degree of empowerment and agency experienced by the patient. It also affects how a patient is supported to adapt to her new circumstances so that life is still meaningful in the face of adversity. Therefore, the nature of the relationship between practitioner and patient impacts ethical decision-making and is integral to person-centred care that is individualised rather than generic.

## Influences on relationship formation

The way in which relationships form between practitioner and patient is unpredictable and subject to myriad different influences. Some of these are internal – bias resulting from preconceptions, the power differential between clinician and patient, the extent to which the clinician is willing to emotionally engage with her patient (mediated by the presence or absence of continuity of care) and the level of congruence between the values and lived experience of practitioner and patient. Hermeneutic injustice is more likely to occur when there is a large gap between the lived experiences of practitioner and patient.

Other influences are external, including the context in which the interaction takes place and other environmental factors. We have argued previously that patterns of interactions in the workplace produce collective values, which influence the behaviour of everyone who works there (Spicer *et al.*, 2021). To extrapolate, the moral positions of clinician and patient are determined by the systems of which they are part. Therefore, the culture of the practice is likely to influence the behaviour of clinicians who work there, the formation of relationships within teams and hence the extent to which epistemic injustice is experienced by patients. The importance of this cannot be underestimated – professional identity formation is shaped by the culture of the workplace (Bleakley, 2006; Webb, 2015).

## Concluding comments

A principlist moral theory assumes that applying a set of principles or ideas will inform the most appropriate course of action. It is based on fixed principles, which are unlikely to be responsive to the essential unpredictability of human interactions. This has direct consequences for interpreting and understanding professional behaviour and the patient experience of healthcare systems. Therefore, assuming that a set of principlist ethical principles will be adequate in protecting patients against epistemic and other forms of injustice can be challenged.

We assert that relational care is fundamental to ameliorating epistemic injustice and that relationships are integral to ethical practice, rather than being distinct. One model of the consultation that recognises this is the four-domain model, featuring the hermeneutic window (Shah *et al.*, 2022). If relational care, which promotes creative capacity, is seen as being an integral part of an ethical approach to healthcare, this has implications for commissioners and providers of primary healthcare. On a practice level, there needs to be a stronger emphasis on protecting the continuity of care with a named clinician or small multiprofessional team. It requires explicit discussion about shared values within the practice team and how these are enacted, as well as increased scrutiny of the quality of the clinician-patient relationship. On a systems level, there is a need for greater community engagement and empowerment to tackle the root causes of disease. These measures are likely to have the additional benefit of leading to more efficient and targeted use of healthcare resources. We propose that epistemic injustice should be more clearly described and articulated and that the framework through which to approach it is relational rather than normative.
